# Comprehensive Screening of Gene Function and Networks by DNA Microarray Analysis in Japanese Patients with Idiopathic Portal Hypertension

**DOI:** 10.1155/2015/349215

**Published:** 2015-10-04

**Authors:** Kohei Kotani, Joji Kawabe, Hiroyasu Morikawa, Tomohiko Akahoshi, Makoto Hashizume, Susumu Shiomi

**Affiliations:** ^1^Department of Nuclear Medicine, Graduate School of Medicine, Osaka City University, 1-4-3 Asahi-machi, Abeno-ku, Osaka 545-8585, Japan; ^2^Department of Hepatology, Graduate School of Medicine, Osaka City University, Osaka 545-8585, Japan; ^3^Department of Disaster and Emergency Medicine, Graduate School of Medical Sciences, Kyushu University, Fukuoka 812-8582, Japan

## Abstract

The functions of genes involved in idiopathic portal hypertension (IPH) remain unidentified. The present study was undertaken to identify the functions of genes expressed in blood samples from patients with IPH through comprehensive analysis of gene expression using DNA microarrays. The data were compared with data from healthy individuals to explore the functions of genes showing increased or decreased expression in patients with IPH. In cluster analysis, no dominant probe group was shown to differ between patients with IPH and healthy controls. In functional annotation analysis using the Database for Annotation Visualization and Integrated Discovery tool, clusters showing dysfunction in patients with IPH involved gene terms related to the immune system. Analysis using network-based pathways revealed decreased expression of adenosine deaminase, ectonucleoside triphosphate diphosphohydrolase 4, ATP-binding cassette, subfamily C, member 1, transforming growth factor-*β*, and prostaglandin E receptor 2; increased expression of cytochrome P450, family 4, subfamily F, polypeptide 3, and glutathione peroxidase 3; and abnormalities in the immune system, nucleic acid metabolism, arachidonic acid/leukotriene pathways, and biological processes. These results suggested that IPH involved compromised function of immunocompetent cells and that such dysfunction may be associated with abnormalities in nucleic acid metabolism and arachidonic acid/leukotriene-related synthesis/metabolism.

## 1. Introduction

Idiopathic portal hypertension (IPH) is characterized by portal hypertension due to obstruction or stenosis of the intrahepatic peripheral portal branches [1, 2]. Patients with IPH present with splenomegaly, anemia, and portal hypertension and are free of obstruction of the extrahepatic portal vein or hepatic vein, hematological disease, parasitic disease, granulomatous liver disease, or congenital hepatic fibrosis. IPH does not usually lead to liver cirrhosis [3–5]. IPH resembles a disease called noncirrhotic portal fibrosis in India or hepatoportal sclerosis/noncirrhotic portal hypertension in Western countries [5, 6]. The etiology of IPH remains unclear. To date, researchers have suggested that IPH may be attributed to intrahepatic peripheral portal vein thrombosis, splenic factors, abnormal autoimmunity, and related factors [7–11].

Our research team has shown that the connective tissue growth factor (CTGF) gene is expressed specifically in patients with IPH. Additionally, we previously reported that patients with IPH exhibit high levels of CTGF in the blood and overexpression of CTGF mRNA in liver tissues [12]. However, in rats with transient overexpression of CTGF induced by recombinant adenovirus, no changes were observed in liver tissues, despite the expression of fibrosis-related genes [13]. Thus, we aimed to examine the specific expression of other genes in patients with IPH.

Recently, comprehensive gene expression analysis with DNA microarrays has been used in a variety of diseases, such as cancer and immunological disease, and has been employed for pathophysiological analysis of these diseases [14–17]. Such analysis enables detection of genes or biological pathways showing significant increases or decreases in expression in the target specimens; this is useful for estimation of the features of the target disease. To the best of our knowledge, no studies have reported comprehensive analysis of gene expression using DNA microarrays in patients with IPH. Thus, in this study, we aimed to perform comprehensive gene expression analysis using DNA microarrays in blood samples from patients with IPH to identify genes showing significant changes in expression. Moreover, we performed biological pathway analysis using the data obtained from DNA microarrays.

## 2. Materials and Methods

### 2.1. Patients

Analysis was conducted using blood samples from four patients with IPH satisfying the IPH diagnostic criteria (prepared under the Intractable Hepatobiliary Disease Program of the Ministry of Health, Labour and Welfare, Japan) and four healthy volunteers as controls [18, 19]. The diagnosis of IPH was based on the following criteria: (1) general findings: one or more cytopenia, normal function or mild dysfunction of the liver, or collateral circulation such as upper gastrointestinal varix, (2) imaging analysis: splenomegaly, atrophy of peripheral parenchyma and enlargement of central parenchyma in the liver, irregular hepatic surface, increase in splenic venous flow, abnormality of intrahepatic peripheral portal branches, mutual binding of hepatic veins, or increase of hepatic venous pressure, (3) pathological findings: collapse or stenosis of peripheral branches of intrahepatic portal veins, fibrosis of splenic sinus, or Gamma-Gandy bodies formation in splenic trabeculae, (4) exclusion of other diseases causing portal hypertension such as cirrhosis, extrahepatic occlusion, Budd-Chiari syndrome, blood disease, parasitic disease, granulomatous liver disease, congenital liver fibrosis, chronic viral hepatitis, and primary biliary cirrhosis, among others. These blood samples had been stored without identifying information at the Sample Storage Center of Kyushu University installed by the Portal Hemodynamics Abnormalities Study Group within the framework of the Intractable Hepatobiliary Disease Program of the Ministry of Health, Labour and Welfare, Japan. The samples were randomly chosen for use in this study. This study was conducted in compliance with the ethical principles of  the Declaration of  Helsinki (1964) and was approved by the ethics committees of Osaka City University and Kyushu University. Informed consent was obtained from all participants.

### 2.2. Sample Adjustment and Microarray Data

All blood samples were stored at −80°C. Total RNA was extracted by ISOGEN-LS (NIPPON GENE Co., Ltd., Tokyo, Japan). RNA levels were measured with a spectrophotometer SmartSpec 3000 (Bio-Rad Laboratories, Inc., Hercules, CA, USA), and the quality of each RNA sample was checked with an Agilent 2100 Bioanalyzer (Agilent Technologies, Inc., Santa Clara, CA, USA). Three samples from patients with IPH and three samples from healthy volunteers (with RNA integrity numbers of ≥ 6) were subjected to microarray analysis. The WT-Ovation Pico RNA Amplification System (NuGEN Technologies, Inc., San Carlos, CA, USA) was employed for cDNA synthesis, conversion into sense chain, fragmentation, and biotin labeling, and a Human Gene ST 1.0 Array (Affymetrix, Inc., Santa Clara, CA, USA) was then used for hybridization of the sense chain cDNA. Array scanning was conducted with a GeneChip 3000 Scanner (Affymetrix, Inc.) to yield image data. The array image data from each sample were converted into files in a format enabling numerical extraction using GeneChip Operating Software (GCOS), a standard data analysis system included with the GeneChip system. Microarray data in this paper have been submitted to National Center for Biotechnology Information (NCBI) Gene Expression Omnibus (GEO) and are accessible through the GEO series accession number GSE69601.

### 2.3. Cluster Analysis

The signal intensity values for data from the three patients with IPH and the three healthy controls were divided by the mean signal intensity values for all six samples, and the quotient was converted into a logarithm. The calculated relative signal intensity values were presented on a heat map and subjected to cluster analysis using Cluster 3.0 software with the following parameters:* k*-means; number of clusters, 12; correlation (uncentered) selected for similarity metric; and number of runs, 1000. The results were visualized with Java TreeView software [20].

### 2.4. Functional Annotation Analysis for Classification of Gene Function

To extract information on the functions of genes identified as differentially expressing using the GeneChip numerical data, the Database for Annotation Visualization and Integrated Discovery (DAVID) tool was used; this database includes the Gene Ontology Database (http://geneontology.org/) and the Kyoto Encyclopedia of Genes and Genomes (http://www.genome.jp/kegg/) [21–23]. For each gene, the ratio between the mean values of the IPH group and the healthy control group, that is, the expression ratio (fold-change), and *p* value obtained using *t*-test were calculated. We used the volcano plot method, which reflects both biological significance and statistical significance. By changing the cut-off value of the expression ratio and *p* value to include gene function terms that may be important, four gene groups were defined, including three groups showing a significant reduction in expression in the presence of IPH (Group A: healthy control/IPH > 2.0, *p* < 0.05; Group B: healthy control/IPH > 1.5, *p* < 0.05; Group C: healthy control/IPH > 1.5, *p* < 0.1) and one group showing significant elevation of expression in the presence of IPH (Group D: IPH/healthy control > 1.5, *p* < 0.1). For each group, functional annotation chart analysis and functional annotation clustering analysis were performed.

### 2.5. Biological Interpretation Using Gene Ontology and Network-Based Pathway Analysis

Gene network analysis was performed using Ingenuity iReport (Ingenuity Systems, http://www.ingenuity.com/, Redwood City, CA, USA). This analysis enabled us to identify differentially expressed genes and molecular interactions for the target disease. On the basis of iReport data, differentially expressed genes were focused using *t*-tests and fold changes, followed by biological interpretation with Ingenuity ontology and canonical pathways. For each pathway, biological process, and disease classification, categories were arranged in the order of involvement rate ranking. Furthermore, relevant genes and networks were also explored by Ingenuity Pathways Analysis (IPA; Ingenuity Systems), with reference to the top 10 significant pathways selected by IPA. Biological interpretation was made from these results.

## 3. Results

### 3.1. Cluster Analysis

**Figure 1:**
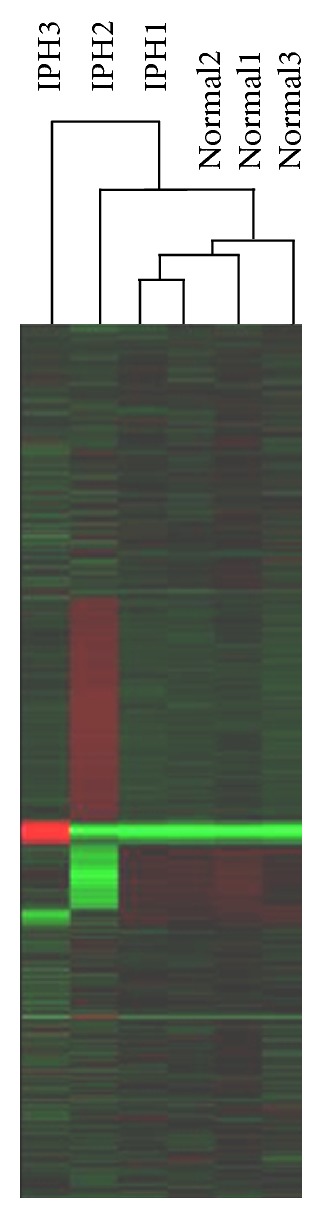
Results of cluster analysis of gene expression data. The dark purple to red regions indicate relative increases in expression, while the dark green to yellow-green regions indicate relative decreases in expression. There were no dominant increases/decreases in gene expression in the IPH group as compared to the healthy control group.

Figure 1 represents the results of cluster analysis. Samples from all three healthy controls showed similar trends (increases or decreases) in gene expression, while samples from the three patients with IPH showed differing trends. No dominant probe group showing a clear difference between the IPH group and the healthy control group was obtained.

### 3.2. Functional Annotation Analysis of Differentially Expressed Genes

**Figure 2:**
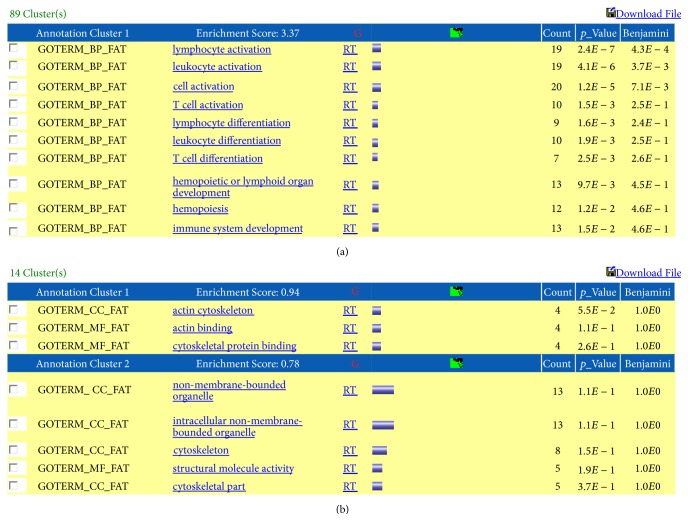
The expression ratio (ratio of the mean in the IPH group to the mean in the healthy control group) was calculated for each gene. Results of functional annotation clustering analysis are given for (a) the group showing significantly reduced expression among patients with IPH (Group C: healthy control/IPH > 1.5, *p* < 0.1) and (b) the group showing significantly increased expression among patients with IPH (Group D: IPH/healthy control > 1.5, *p* < 0.1). (a) Group C had the highest enrichment score (3.37), and its cluster included many gene terms related to the immune system, such as “lymphocyte activation” and “leukocyte activation.” (b) In Group D, the enrichment score was low (0.94), and no gene function terms were found to be specifically increased in the presence of IPH.

Table 1 shows the number of charts extracted with the functional annotation chart and the number of clusters extracted by functional annotation clustering. The number of charts was smallest (three) in Group D. Among all groups, clusters for Group C (exhibiting the highest enrichment score (3.37)) contained many gene terms related to the immune system, including “lymphocyte activation” and “leukocyte activation” (Figure 2(a)). This finding suggested that immunological abnormalities were involved in IPH. In Group D, the enrichment score was low (0.94), and no gene function terms were found to be specifically increased in the presence of IPH (Figure 2(b)).

### 3.3. Biological Interpretation Using Network-Based Pathway Analysis

**Figure 3:**
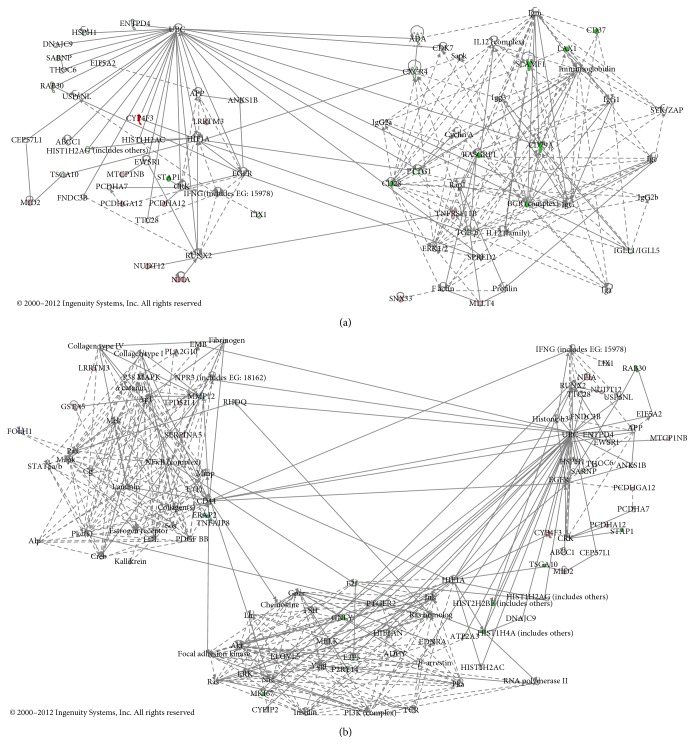
IPA network illustration of the relationships among gene groups showing significantly increased/decreased expression in the presence of IPH. Genes colored pink or red exhibited increased expression, while genes colored green exhibited reduced expression. The straight lines indicate direct relationships among genes, while broken lines indicate indirect relationships. (a) Illustration of the first selected pathway combined with the second pathway. (b) Illustration of the first pathway combined with the third and fifth pathways. Similar to the results from Ingenuity iReport analysis, reduced expression of ENTPD4, ABCC1, ADA, and TGF-*β* and increased expression of CYP4F3 were observed inside the IPA network.

Supplementary Table 1 in Supplementary Material available online at http://dx.doi.org/10.1155/2015/349215 lists all genes selected on the basis of the iReport. For each pathway, biological process, and disease classification, we extracted categories in the top 25 positions of the involvement rate ranking (Tables 2 [Table tab3]–4). The highest ranked target in pathway classification was reduced adenosine deaminase (ADA) expression. Additionally, the target at rank 21 in the pathway classification was abnormal purine metabolism. Targets 8 and 15 in pathway classification and targets 1, 2, and 11 in biological process classification were associated with abnormalities in the synthesis/metabolism of arachidonic acid- (AA-) prostaglandin- (PG-) leukotriene (LT). Expression levels of cytochrome P450, family 4, subfamily F, polypeptide 3 (CYP4F3), and glutathione peroxidase 3 (GPX3), which are involved in leukotriene B4 (LTB4) metabolism, were increased, while the expression levels of the PG E receptor 2 (PTGER2) were reduced. Furthermore, target 22 in pathway classification and target 13 in disease classification were associated with abnormal endothelin (ET) signals. The expression of the ET receptor type A (EDNRA) was reduced, while the expression of natriuretic peptide receptor 3 (NPR3) was increased. Additionally, the expression levels of cluster of differentiation 44 (CD44) and transforming growth factor (TGF)-*β* were reduced.  Figure 3(a) shows combination of the first pathway with the second pathway, and Figure 3(b) shows combination of the first pathway with the third and fifth pathways; each of these plots was prepared with reference to the top 10 significant pathways selected by IPA. Reduced expression of the ectonucleoside triphosphate diphosphohydrolase 4 (ENTPD4), ATP-binding cassette, subfamily C, member 1 (ABCC1), ADA, and TGF-*β* and increased expression of CYP4F3 were noted. Thus, the results from the iReport were also observed within the IPA network.

## 4. Discussion

IPH is a rare disease. In Japan, IPH was officially added to the list of specific intractable diseases covered by the medical expense subsidy program in January of 2015. IPH has been reported to be associated with autoimmune abnormalities [10, 11, 24]; however, the exact pathophysiology of IPH remains unclear. In this study, we attempted, for the first time, to explore the features of IPH through comprehensive gene analysis with DNA microarrays.

The results of cluster analysis showed that there were no dominant increases/decreases in gene expression in the IPH group as compared to the healthy control group. IPH has been conventionally viewed as a syndrome observed in individuals free of any other disease that can elevate portal pressure. The diagnosis of IPH generally involves ruling out other potential diagnoses, and the pathophysiological features of IPH are not uniform. Thus, these characteristics of IPH may explain the above-mentioned results.

In the analysis of functional annotation charts, many genes showed significantly reduced expression in the IPH group, but only three charts in the IPH group showed significantly increased expression. In functional annotation clustering analysis, the enrichment score of the Group C cluster was the highest (3.37), and this cluster contained many gene terms related to the immune system. Coupled with the observation that some particular trends are found when the enrichment score rises to 2.0 or higher, our data suggested that immunological abnormalities are involved in the pathophysiology of IPH. We also found that the enrichment score in Group D was relatively low (0.94), with no gene function terms showing increased expression in the presence of IPH.

We attempted further clarification of the IPH pathophysiology through network analysis. First, canonical pathway analysis was conducted using the Ingenuity iReport. In pathway classification, reduced expression of ADA was noted. Severe immunodeficiency has been shown to be induced by accumulation of hazardous adenosine metabolites resulting from ADA defects [25, 26]. Thus, reduced expression of ADA may cause induction of autoimmune disease from immunodeficiency, resulting in attack of specific organs. This theory is consistent with the view that immune system abnormalities, particularly suppressor T-cell dysfunction and accompanying autoimmunity, are candidate factors responsible for IPH [24, 27, 28]. ADA dysfunction has also been shown to affect liver differentiation and function [29–31]. It is likely that reduced ADA activity serves as a core factor, and combination of ADA with autoimmunity-dependent injury and direct liver injury leads to manifestation of IPH. In this analysis, abnormal purine metabolism was selected. Based on this connection, ADA deficiency induces accumulation of intermediate metabolites due to abnormal nucleic acid metabolism, thereby exerting cytotoxic effects and disrupting lymphocyte differentiation, leading to onset of severe immunodeficiency [32]. Similar to the effects of ADA deficiency, patients with IPH also have abnormal systemic nucleic acid metabolism as a primary feature; this may lead to induction of abnormal lymphocyte differentiation and abnormal function.

The results of the current study showed that the expression levels of CYP4F3 and GPX3, which are known to be involved in degradation of LTB4 and the LTB4 precursor 5-HPETE, were increased. In contrast, the expression of PTGER2 was decreased. LTB4 is known to activate neutrophils [33], and PGE2 is known to have vasodilatory effects [34]. These results may be viewed as compensatory reactions, corresponding to the increased production of LTB4 and PGE2; elevation of these components in the blood may compensate for leukocyte dysfunction and increased blood pressure caused by other factors. Furthermore, analysis of ET signals revealed reduced EDNRA expression and increased NPR3 expression. Decreased expression of the ET receptor leads to vascular contraction [35], while increased expression of NPR3 leads to vascular dilation [36]. Taken together, these findings suggest that NPR3 expression increases as a compensatory reaction to vascular contraction, possibly causing dilation of blood vessels.

When viewed as a whole, the expression of CD44 and TGF-*β* was reduced. CD44 is known to induce T-cell homing and activate T-helper 1 (Th1) cells, T-helper 2 (Th2) cells, cytotoxic T lymphocytes (CTLs), and natural killer (NK) cells and is thus involved in stem cell proliferation and differentiation [37–39]. TGF-*β* suppresses the proliferation of blood cells and induces the proliferation and activation of mesenchymal cells involved in blood cell differentiation [40, 41]. Thus, in patients with IPH, immune system responses and the differentiation-inducing environment appear to be disturbed.

Furthermore, we explored the network pathway involved in IPH using IPA network analysis. Although the IPA network results did not reveal any additional finding, many genes selected by iReport were also observed in the high-ranked IPA pathways, thus strongly supporting the iReport results.

Taken together, these findings suggest that abnormal systemic nucleic acid metabolism is the first factor involved in the onset of IPH and that this abnormality induces abnormal differentiation and function of immunocompetent cells, although our results are preliminary and require further analysis. In this context, abnormalities in AA-related synthesis/metabolism and ET signals can be viewed as compensatory reactions of blood cells corresponding to changes in the external environment of blood cells, for example, increases/decreases in the levels of certain factors in the serum.

The present study enrolled four IPH patients and three of four samples were used for gene analyses. We cannot deny the possibility that our results did not cover the patterns of whole IPH patients because the sample number was small. In addition, we conducted comprehensive gene expression analysis of blood samples. Splenomegaly is a characteristic symptom in patients with IPH and is often accompanied by histological abnormalities, such as splenic sinus hyperplasia and increased collagen fibers. Thus, analysis of splenic tissue specimens may be useful for clarifying the pathophysiology of IPH. However, unlike the liver, in which percutaneous biopsy is relatively easy, the spleen is difficult to be biopsied or resected in healthy individuals and in patients with IPH during routine clinical practice. To date, no reports have described comprehensive gene expression analysis in patients with IPH. Therefore, additional genetic studies are needed to confirm the biological roles of the molecular pathology of IPH determined in the present study. The identification of genetic abnormalities in IPH may lead to the development of genetic therapy.

## 5. Conclusion

Blood samples from patients with IPH and healthy individuals as controls were subjected to comprehensive gene analysis with DNA microarrays. In cluster analysis, we did not observe any dominant probe groups showing clear differences between the IPH group and the healthy control group. In functional annotation analysis, the cluster of compromised function among patients with IPH included many gene terms primarily related to the immune system. Network analysis revealed suppression of immune function, abnormal nucleic acid metabolism, and abnormal AA-related synthesis/metabolism. These results suggested that abnormal systemic nucleic acid metabolism was a key factor involved in the onset of IPH and that this abnormality induced abnormal differentiation and function in immunocompetent cells. The abnormalities in AA-related synthesis/metabolism and ET signals may represent compensatory reactions in blood cells, corresponding to changes in the external environment of blood cells, such as increases/decreases in the levels of certain factors in the serum.

## Supplementary Material

Supplementary Table 1: All genes showing significantly increased or decreased expression in patients with IPH selected on the basis of the iReport.

## Figures and Tables

**Table 1:** Number of charts and clusters extracted by functional annotation analysis.

	Gene expression ratio	Number of charts	Number of clusters
Group A	Control/IPH > 2.0, *p* < 0.05	28	17
Group B	Control/IPH > 1.5, *p* < 0.05	124	45
Group C	Control/IPH > 1.5, *p* < 0.1	167	89
Group D	IPH/Control > 1.5, *p* < 0.1	3	14

**Table 2:** Top 25 canonical pathways associated with IPH in Ingenuity iReport.

Number	Pathways	DEGs	*p* value	Genes
1	Primary immunodeficiency signaling	4	0.000120085	ADA, CD79A, IGHD, IGLL1/IGLL5
2	Altered T-cell and B-cell signaling in rheumatoid arthritis	4	0.000772903	CD28, SLAMF1, TGFB1, CD79A
3	T-helper cell differentiation	3	0.004440048	CD28, TGFB1, TNFRSF11B
4	Cyclins and cell cycle regulation	3	0.006251755	TGFB1, CDK7, E2F5
5	B-cell development	2	0.010284999	CD79A, IGHD
6	Leukocyte extravasation signaling	4	0.013183985	CXCR4, CD44, MLLT4, MMP12
7	Atherosclerosis signaling	3	0.019146088	TGFB1, CXCR4, PLA2G10
8	Arachidonic acid metabolism	3	0.021798453	GPX3, PLA2G10, CYP4F3
9	Colorectal cancer metastasis signaling	4	0.027547337	RHOQ, TGFB1, PTGER2, MMP12
10	Aryl hydrocarbon receptor signaling	3	0.028201448	TGFB1, NFIA, GSTA5
11	Hepatic fibrosis/hepatic stellate cell activation	3	0.029806364	TGFB1, EDNRA, TNFRSF11B
12	Glioma invasiveness signaling	2	0.030646264	RHOQ, CD44
13	Glutathione metabolism	2	0.031643803	GPX3, GSTA5
14	Cell cycle: G1/S checkpoint regulation	2	0.03367696	TGFB1, E2F5
15	Eicosanoid signaling	2	0.03367696	PLA2G10, PTGER2
16	Induction of apoptosis by HIV1	2	0.03367696	CXCR4, TNFRSF11B
17	Hypoxia signaling in the cardiovascular system	2	0.037891658	UBE2D2, HIF1AN
18	Tight junction signaling	3	0.038540606	TGFB1, MLLT4, TNFRSF11B
19	Germ cell-sertoli cell junction signaling	3	0.039164578	RHOQ, TGFB1, MLLT4
20	Mitotic roles of Polo-like kinase	2	0.040070987	TGFB1, PTTG1
21	Purine metabolism	4	0.043235613	ENTPD4, ABCC1, ADA, SEPT1
22	Endothelin-1 signaling	3	0.046366635	PLA2G10, EDNRA, PTGER2
23	Regulation of IL-2 expression in activated and anergic T Lymphocytes	2	0.05532422	CD28, TGFB1
24	Chronic myeloid leukemia signaling	2	0.073630475	TGFB1, E2F5
25	HMGB1 signaling	2	0.075004378	RHOQ, TNFRSF11B

Underlined genes indicate increases in expression. Not underlined genes indicate decreases in expression.

DEGs: differentially expressed genes.

**Table 3 tab3:** Top 25 biological processes associated with IPH in Ingenuity iReport.

Number	Biological process	DEGs	*p* value	Genes
1	Synthesis of leukotriene B4	4	6.76164*E* − 06	TGFB1, ABCC1, ADA, PTGER2
2	Synthesis of leukotriene	5	8.36612*E* − 06	TGFB1, PLA2G10, ABCC1, ADA, PTGER2
3	Quantity of pro-B lymphocytes	5	9.10676*E* − 06	CD28, CXCR4, CD79A, IGLL1/IGLL5, TNFRSF11B
4	T-cell migration	8	1.0515*E* − 05	CD28, TGFB1, CXCR4, GNLY, RASGRP1, PLA2G10, ABCC1, CD44
5	Arrest in cell cycle progression of keratinocyte cancer cell lines	2	2.27598*E* − 05	TGFB1, MELK
6	Function of leukocytes	12	2.82837*E* − 05	CD28, SLAMF1, TGFB1, CXCR4, RASGRP1, PLA2G10, ABCC1, ADA, CD44, PTGER2, MMP12, LAX1
7	Transcytosis of HIV-1	2	6.80667*E* − 05	CXCR4, CD79A
8	Cell movement of hairy leukemia cells	2	6.80667*E* − 05	TGFB1, CD44
9	Invasion of keratinocyte cancer cell lines	2	6.80667*E* − 05	TGFB1, PTGER2
10	Signaling of T lymphocytes	3	8.23941*E* − 05	CD28, SLAMF1, CD44
11	Metabolism of eicosanoid	7	0.000109678	TGFB1, PLA2G10, ABCC1, ADA, CYP4F3, EDNRA, PTGER2
12	Reorganization of membrane rafts	2	0.000135709	CD28, CD44
13	Response of memory T lymphocytes	2	0.000135709	CD28, TGFB1
14	Fusion of leukocytes	3	0.000153367	CXCR4, CD44, TNFRSF11B
15	Lymphopoiesis of cells	3	0.000153367	TGFB1, CXCR4, CD44
16	Migration of mammary tumor cells	3	0.000153367	TGFB1, CXCR4, CD44
17	Cell viability of leukocytes	7	0.000162768	CD28, TGFB1, CXCR4, CD44, LAX1, CD79A, TNFRSF11B
18	Transmigration of mononuclear leukocytes	4	0.000170867	CD28, TGFB1, CXCR4, CD44
19	Lymphopoiesis	4	0.000182632	TGFB1, CXCR4, CD44, IGLL1/IGLL5
20	Adhesion of prostate cancer cell lines	3	0.000200182	TGFB1, CXCR4, CD44
21	Invasion of breast cell lines	3	0.000200182	TGFB1, CXCR4, PTGER2
22	TH1 immune response of naive T lymphocytes	2	0.000225478	CD28, TGFB1
23	Adhesion of hyaluronic acid	2	0.000225478	TGFB1, CD44
24	Morphology of cardiovascular tissue	4	0.000235591	TGFB1, CXCR4, CD44, TNFRSF11B
25	Migration of tumor cell lines	10	0.000252381	TNFAIP8, TGFB1, CXCR4, RASGRP1, CDK7, PTTG1, SERPINA5, CD44, E2F5, MLLT4

Underlined genes indicate increases in expression. Not underlined genes indicate decreases in expression.

DEGs: differentially expressed genes.

**Table 4:** Top 25 diseases associated with IPH in Ingenuity iReport.

Number	Disease	DEGs	*p* value	Genes
1	Immunodeficiency	6	1.805*E* − 06	CD28, CXCR4, RASGRP1, ADA, CD79A, IGLL1/IGLL5
2	Leprosy	5	5.11653*E* − 05	CD28, SLAMF1, TGFB1, CD79A, IGLL1/IGLL5
3	Transcytosis of HIV-1	2	6.80667*E* − 05	CXCR4, CD79A
4	Genital tumor	15	0.000101439	CXCR4, CDK7, FOLH1, STAP1, MKI67, GPX3, ABCA8, TGFB1, ABCC1, SERPINA5, CD44, EDNRA, PTGER2, TNFRSF11B, PEG3
5	Agammaglobulinemia	2	0.000135709	CD79A, IGLL1/IGLL5
6	Metastasis of tumor	5	0.000161747	CD28, TGFB1, CXCR4, CD44, PTGER2
7	Digestive organ tumor	20	0.000177474	ERAP2, HIST1H4A, CXCR4, CDK7, PTTG1, FOLH1, TPD52L1, MELK, MKI67, IGLL1/IGLL5, GPX3, TGFB1, OIT3, ABCC1, C7, E2F5, CD44, MMP12, PEG3, TNFRSF11B
8	Colorectal cancer	14	0.000211188	ERAP2, HIST1H4A, CXCR4, PTTG1, CDK7, FOLH1, TPD52L1, MELK, MKI67, GPX3, TGFB1, CD44, TNFRSF11B, PEG3
9	Tuberculoid leprosy	3	0.000255384	CD28, SLAMF1, CD79A
10	Metastasis	10	0.000432136	CD28, ERAP2, TGFB1, CXCR4, PTTG1, CD44, MLLT4, PTGER2, PEG3, TNFRSF11B
11	Granulocyte colony stimulating factor-induced psoriasiform dermatitis	2	0.000470563	TGFB1, MKI67
12	Rectum cancer	3	0.0005718	GPX3, CD44, MKI67
13	Vascular disease	11	0.00075445	NPR3, SERPINA5, MARC1, TGFB1, EDNRA, PTGER2, CD44, MMP12, CD28, MKI67, CXCR4
14	Infection by bacteria	8	0.00090366	CD28, SLAMF1, TGFB1, SERPINA5, CD44, MMP12, CD79A, IGLL1/IGLL5
15	Replication of HIV-1	4	0.00121203	CD28, TGFB1, CXCR4, PLA2G10
16	Pulmonary hypertension	3	0.001305595	NPR3, CXCR4, EDNRA
17	Prostatic tumor	9	0.001615688	GPX3, TGFB1, CXCR4, FOLH1, CD44, EDNRA, STAP1, MKI67, TNFRSF11B
18	Uterine serous papillary cancer	6	0.001802487	ABCA8, P2RY14, PTTG1, C7, EDNRA, PEG3
19	Fibrosis of lung	5	0.001814056	TGFB1, PLA2G10, ADA, EDNRA, MMP12
20	Cancer	33	0.001947463	ERAP2, HIST2H2BE, PTTG1, FOLH1, TPD52L1, MLLT4, MELK, MKI67, CD79A, IGLL1/IGLL5, GPX3, CD28, SARNP, TGFB1, ABCC1, C7, E2F5, ADA, MMP12, TNFRSF11B, PEG3, HIST1H4A, CXCR4, CDK7, STAP1, ABCA8, P2RY14, RASGRP1, OIT3, SERPINA5, CD44, EDNRA, PTGER2
21	Endometrial carcinoma	8	0.002077475	GPX3, ABCA8, P2RY14, TGFB1, PTTG1, C7, EDNRA, PEG3
22	Neuroblastoma	3	0.002212756	CXCR4, ABCC1, CD44
23	Edema of tissue	2	0.002294982	TGFB1, SPRED2
24	Carcinoma	28	0.002351502	ERAP2, HIST2H2BE, PTTG1, FOLH1, TPD52L1, MELK, MKI67, IGLL1/IGLL5, GPX3, TGFB1, ABCC1, C7, E2F5, MMP12, TNFRSF11B, PEG3, HIST1H4A, CXCR4, CDK7, STAP1, ABCA8, P2RY14, RASGRP1, OIT3, SERPINA5, CD44, EDNRA, PTGER2
25	Polyuria	3	0.002583567	NPR3, PTTG1, ABCC1

Underlined genes indicate increases in expression. Not underlined genes indicate decreases in expression.

DEGs: differentially expressed genes.
