# Supplementary material for: Comprehensive Screening of Gene Function and Networks by DNA Microarray Analysis in Japanese Patients with Idiopathic Portal Hypertension
Source: Mediators Inflamm. 2015 Oct 4;2015:349215. doi: 10.1155/2015/349215 (PMC4609492; doi:10.1155/2015/349215)
Supplement: Supplementary file 1 — Supplementary Table 1: All genes showing significantly increased or decreased expression in patients with IPH selected on the basis of the iReport. [file 349215.f1.pdf]

SUPPLEMENTARY TABLE 1: All 113 genes showing significantly increased or decreased expression in patients with IPH on the basis of the iReport

**Symbol**	**Fold Change**	**Molecular Function**	**Location**	**Pathways**	**Processes**	**Diseases**
CYP4F3: cytochrome P450, family 4, subfamily F, polypeptide 3	7.012	enzyme	Cytoplasm	1	3	1
C7: complement component 7	2.666	other	Extracellular Space	2	0	10
FOLH1: folate hydrolase (prostate-specific membrane antigen) 1	2.631	peptidase	Plasma Membrane	0	2	8
ABCA8: ATP-binding cassette, sub-family A (ABC1), member 8	2.393	transporter	Plasma Membrane	0	0	10
OIT3: oncoprotein induced transcript 3	2.376	other	Nucleus	0	0	4
NPR3 (includes EG:18162): natriuretic peptide receptor C/guanylate cyclase C (atrionatriuretic peptide receptor C)	2.253	G-protein coupled receptor	Plasma Membrane	2	3	5
SERPINA5: serpin peptidase inhibitor, clade A (alpha-1 antiproteinase, antitrypsin),	2.245	other	Extracellular Space	1	8	11

member 5						
PEG3: paternally expressed 3	2.235	kinase	Nucleus	0	0	13
PCDHGB5: protocadherin gamma subfamily B, 5	2.184	other	Unknown	0	0	0
TPD52L1: tumor protein D52-like 1	2.181	other	Cytoplasm	0	2	5
MARC1: mitochondrial amidoxime reducing component 1	2.087	enzyme	Cytoplasm	1	0	2
MTCP1NB: mature T-cell proliferation 1 neighbor	2.057	other	Cytoplasm	0	0	0
PAIP1: poly(A) binding protein interacting protein 1	2.053	translation regulator	Cytoplasm	2	0	0
PLA2G10: phospholipase A2, group X	1.984	enzyme	Cytoplasm	17	39	6
LRRTM3: leucine rich repeat transmembrane neuronal 3	1.966	other	Unknown	0	0	0
PCDHGC4: protocadherin gamma	1.858	other	Unknown	0	0	0

subfamily C, 4						
PCDHGA12: protocadherin gamma subfamily A, 12	1.789	other	Plasma Membrane	0	0	0
PCDHA12: protocadherin alpha 12	1.777	other	Plasma Membrane	0	0	0
NFIA: nuclear factor I/A	1.772	transcription regulator	Nucleus	1	7	1
OR4N4: olfactory receptor, family 4, subfamily N, member 4	1.706	G-protein coupled receptor	Plasma Membrane	0	0	0
SPRED2: sprouty-related, EVH1 domain containing 2	1.705	cytokine	Extracellular Space	0	2	1
TNFRSF11B: tumor necrosis factor receptor superfamily, member 11b	1.684	transmembrane receptor	Plasma Membrane	19	40	28
MLLT4: myeloid/lymphoid or mixed-lineage leukemia (trithorax homolog, Drosophila); translocated to, 4	1.635	other	Nucleus	4	8	4
MANSC1: MANSC domain containing 1	1.63	other	Unknown	0	0	0

SNX33: sorting nexin 33	1.586	other	Cytoplasm	0	1	0
MID2: midline 2	1.576	other	Cytoplasm	0	0	0
YBEY: ybeY metallopeptidase (putative)	1.544	other	Cytoplasm	0	0	0
SCRN3: secernin 3	1.539	other	Unknown	0	0	0
TPRG1L: tumor protein p63 regulated 1-like	1.537	other	Cytoplasm	0	0	0
IPP: intracisternal A particle-promoted polypeptide	1.536	other	Cytoplasm	0	1	0
LINC00238: long intergenic non-protein coding RNA 238	1.533	other	Unknown	0	0	0
ZMYND12: zinc finger, MYND-type containing 12	1.531	other	Unknown	0	0	0
GPX3: glutathione peroxidase 3 (plasma)	1.529	enzyme	Extracellular Space	2	0	14
C1QTNF7: C1q and tumor necrosis factor related protein 7	1.52	other	Extracellular Space	0	0	0

LOC286359: uncharacterized LOC286359	1.513	other	Unknown	0	0	0
GSTA5: glutathione S-transferase alpha 5	1.504	enzyme	Cytoplasm	6	0	0
NUDT12: nudix (nucleoside diphosphate linked moiety X)-type motif 12	1.504	phosphatase	Cytoplasm	1	0	0
TGFB1 (includes EG:21803): transforming growth factor, beta 1	-1.501	growth factor	Extracellular Space	33	239	46
P2RY14: purinergic receptor P2Y, G-protein coupled, 14	-1.51	G-protein coupled receptor	Plasma Membrane	2	1	7
TPTE2P2: transmembrane phosphoinositide 3-phosphatase and tensin homolog 2 pseudogene 2	-1.516	other	Unknown	0	0	0
QTRTD1: queuine tRNA-ribosyltransferase domain containing 1	-1.521	other	Cytoplasm	0	0	0
SARNP: SAP domain containing ribonucleoprotein	-1.543	other	Nucleus	0	0	2

UBE2D2: ubiquitin-conjugating enzyme E2D 2	-1.544	enzyme	Cytoplasm	2	0	0
RHOQ: ras homolog family member Q	-1.546	enzyme	Plasma Membrane	23	1	0
CDK7: cyclin-dependent kinase 7	-1.548	kinase	Nucleus	11	5	8
ADA: adenosine deaminase	-1.553	enzyme	Cytoplasm	2	47	8
SEPT1: septin 1	-1.558	enzyme	Cytoplasm	3	0	0
FOXRED2: FAD-dependent oxidoreductase domain containing 2	-1.562	other	Cytoplasm	0	0	0
ENTPD4: ectonucleoside triphosphate diphosphohydrolase 4	-1.566	enzyme	Cytoplasm	2	2	1
HSD17B7P2: hydroxysteroid (17-beta) dehydrogenase 7 pseudogene 2	-1.577	other	Unknown	0	0	0
SLC38A11: solute carrier family 38, member 11	-1.589	transporter	Unknown	0	0	0
ELOVL5: ELOVL fatty acid elongase 5	-1.601	enzyme	Cytoplasm	0	3	0

ELMOD3: ELMO/CED-12 domain containing 3	-1.621	other	Unknown	0	0	0
EMB: embigin	-1.623	other	Plasma Membrane	0	0	0
ATP2A3: ATPase, Ca++ transporting, ubiquitous	-1.63	transporter	Cytoplasm	5	0	0
PRO0611: PRO0611 protein	-1.637	other	Unknown	0	0	0
E2F5: E2F transcription factor 5, p130-binding	-1.637	transcription regulator	Nucleus	12	12	4
ABCC1: ATP-binding cassette, sub-family C (CFTR/MRP), member 1	-1.647	transporter	Plasma Membrane	3	48	13
TMEM120B: transmembrane protein 120B	-1.657	other	Unknown	0	0	0
EDNRA: endothelin receptor type A	-1.658	transmembrane receptor	Plasma Membrane	4	33	16
SPDYE3: speedy homolog E3 (Xenopus laevis)	-1.659	other	Unknown	0	0	0
C11orf39: chromosome 11 open reading	-1.665	other	Unknown	0	0	0

frame 39						
PTGER2: prostaglandin E receptor 2 (subtype EP2), 53kDa	-1.675	G-protein coupled receptor	Plasma Membrane	6	31	17
MGC27345: uncharacterized protein MGC27345	-1.769	other	Unknown	0	0	0
SNORD80: small nucleolar RNA, C/D box 80	-1.789	other	Unknown	0	0	0
SNORD36A: small nucleolar RNA, C/D box 36A	-1.79	other	Unknown	0	0	0
CD44 (includes EG:100330801): CD44 molecule (Indian blood group)	-1.797	enzyme	Plasma Membrane	7	117	28
LRRN1: leucine rich repeat neuronal 1	-1.827	other	Unknown	0	0	0
HIF1AN: hypoxia inducible factor 1, alpha subunit inhibitor	-1.849	enzyme	Nucleus	1	0	0
TNFAIP8: tumor necrosis factor,	-1.851	other	Cytoplasm	0	9	0

alpha-induced protein 8						
CYFIP2: cytoplasmic FMR1 interacting protein 2	-1.852	other	Cytoplasm	2	0	0
IGHD: immunoglobulin heavy constant delta	-1.868	other	Extracellular Space	3	0	0
LIX1: Lix1 homolog (chicken)	-1.902	other	Unknown	0	0	0
MELK: maternal embryonic leucine zipper kinase	-1.962	kinase	Cytoplasm	0	5	5
IGLL1/IGLL5: immunoglobulin lambda-like polypeptide 1	-1.964	other	Plasma Membrane	1	21	9
HIST1H4A (includes others): histone cluster 1, H4a	-1.971	other	Nucleus	0	1	6
SLC4A7: solute carrier family 4, sodium bicarbonate cotransporter, member 7	-1.971	transporter	Plasma Membrane	0	2	0
HSPH1: heat shock 105kDa/110kDa protein	-1.989	other	Cytoplasm	2	4	0

1						
TSGA10: testis specific, 10	-2.017	other	Cytoplasm	0	0	0
WASH3P: WAS protein family homolog 3 pseudogene	-2.033	other	Plasma Membrane	0	0	0
MMP12: matrix metallopeptidase 12 (macrophage elastase)	-2.036	peptidase	Extracellular Space	4	21	12
RAB30: RAB30, member RAS oncogene family	-2.045	enzyme	Cytoplasm	0	0	0
SNORD44: small nucleolar RNA, C/D box 44	-2.062	other	Unknown	0	0	0
SNORD53: small nucleolar RNA, C/D box 53	-2.122	other	Unknown	0	0	0
APOBEC3F: apolipoprotein B mRNA editing enzyme, catalytic polypeptide-like 3F	-2.126	enzyme	Cytoplasm	1	0	1

SNORA24: small nucleolar RNA, H/ACA box 24	-2.137	other	Unknown	0	0	0
HIST1H2AG (includes others): histone cluster 1, H2ag	-2.14	other	Nucleus	0	0	0
RASGRP1: RAS guanyl releasing protein 1 (calcium and DAG-regulated)	-2.16	other	Cytoplasm	3	28	7
POF1B: premature ovarian failure, 1B	-2.191	other	Plasma Membrane	0	0	0
TBC1D3P2: TBC1 domain family, member 3 pseudogene 2	-2.198	other	Unknown	0	0	0
CD28: CD28 molecule	-2.208	other	Plasma Membrane	17	101	14
HIST1H2BM: histone cluster 1, H2bm	-2.3	other	Nucleus	0	0	0
MKI67: antigen identified by monoclonal antibody Ki-67	-2.314	other	Nucleus	0	2	16
PTTG1: pituitary tumor-transforming 1	-2.441	transcription regulator	Nucleus	1	20	15
HIST2H2BE (includes others): histone	-2.452	other	Nucleus	0	0	4

cluster 2, H2be						
SNORA70E: small nucleolar RNA, H/ACA box 70E (retrotransposed)	-2.487	other	Unknown	0	0	0
CXCR4: chemokine (C-X-C motif) receptor 4	-2.512	G-protein coupled receptor	Plasma Membrane	8	87	29
LOC100507433: uncharacterized LOC100507433	-2.526	other	Unknown	0	0	0
ZNF439: zinc finger protein 439	-2.55	other	Nucleus	0	0	0
SLAMF1: signaling lymphocytic activation molecule family member 1	-2.564	transmembrane receptor	Plasma Membrane	1	18	4
LAX1: lymphocyte transmembrane adaptor 1	-2.583	other	Cytoplasm	0	22	0
DEFB131: defensin, beta 131	-2.608	other	Unknown	0	0	0
SNORA50: small nucleolar RNA, H/ACA box 50	-2.676	other	Unknown	0	0	0

FCRL2: Fc receptor-like 2	-2.807	other	Unknown	0	0	0
CD37: CD37 molecule	-2.834	other	Plasma Membrane	0	11	0
CD79A: CD79a molecule, immunoglobulin-associated alpha	-2.918	transmembrane receptor	Plasma Membrane	10	19	9
GNLY: granulysin	-2.991	other	Cytoplasm	0	28	1
PARP15: poly (ADP-ribose) polymerase family, member 15	-3.068	other	Unknown	1	0	0
GOLGA8A/GOLGA8B: golgin A8 family, member A	-3.418	other	Cytoplasm	0	0	0
STAP1: signal transducing adaptor family member 1	-3.816	other	Cytoplasm	0	1	6
TMEM156: transmembrane protein 156	-3.847	other	Unknown	0	0	0
ERAP2: endoplasmic reticulum aminopeptidase 2	-5.332	peptidase	Cytoplasm	0	1	6
